# Satellite Lesions in Congenital Melanocytic Nevi—Time for a Change of Name

**DOI:** 10.1111/j.1525-1470.2010.01199.x

**Published:** 2011-03-21

**Authors:** VERONICA KINSLER

**Affiliations:** Paediatric Dermatology, Great Ormond Street Hospital for Children NHS Trust/Institute of Child HealthLondon, UK

## Abstract

**Abstract:**

The term “satellite lesions” is used in many conditions in dermatology, generally to describe smaller lesions near the edges of a principal lesion. An online medical dictionary gives the definition “a smaller lesion accompanying a main one and situated nearby,” and this can apply both macroscopically and microscopically. The implication is that the smaller lesions have spread from the parent lesion. Given this definition and usual understanding of the term its use is not apt in the case of congenital melanocytic nevi (CMN). In the vast majority of cases where the patient is said to have satellite lesions these are not restricted to the area around or near the edge of the principal lesion, and are not necessarily significantly smaller. It seems likely that the early use of the term in this condition is an adaptation of the established and correct use in the context of melanoma. Not only is the term not apt clinically but has no known etiological basis. This leaves us with the question of what to call these lesions in cases of CMN. This proposal is to categorise cases into single or multiple CMN, where multiple is 2 or more nevi of any size at birth. An accurate count or good estimate of the number of lesions at birth should also be recorded, and the largest lesion classified as usual with respect to projected adult size.

The term ‘satellite lesions’ is used in many conditions in dermatology, generally to describe smaller lesions near the edges of a principal lesion. An online medical dictionary gives the definition ‘a smaller lesion accompanying a main one and situated nearby’ (1), and this can apply both macroscopically and microscopically. The implication is that the smaller lesions have spread from the parent lesion.

Given this definition and usual understanding of the term its use is not apt in the case of congenital melanocytic nevi (CMN). In the vast majority of cases where the patient is said to have satellite lesions, these are not restricted to the area around or near the edge of the principal lesion, and are not necessarily significantly smaller. It seems most likely that the use of the term in this condition is an adaptation of the earlier established and still correct use in the context of melanoma (2), although exactly how its use first arose is not clear. It appears to have come into common usage in the literature around the 1980s.

Furthermore, to think of the ‘satellites’ as somehow subordinate to the largest CMN is unhelpful in elucidating their etiology. They are melanocytic nevi in their own right and therefore probably represent separate genetic events, at least at the somatic level. A single CMN without any ‘satellites’ suggests only one genetic event, independent of the size of the CMN; a larger single CMNs could simply be attributable to that single event occurring earlier in fetal development. My view is that the presence of just one ‘satellite’ is highly significant as it represents two genetic events in one individual. If the incidence of a small CMN is around 1 in 100 births, then the probability of two small CMN arising by chance in one individual is 1 in 10,000, and for 3 this rises to 1 in 1,000,000. More likely is that the presence of more than one CMN is the result of susceptibility to that event. This would also explain why giant CMNs are strongly associated with ‘satellites’, as any genetic susceptibility would make an early event more likely.

This leaves us with the question of what to call these lesions in cases of CMNs. I propose that we simply categorize cases into single or multiple CMNs, where multiple is two or more nevi of any size at birth. An accurate count or good estimate of the number and range of size of lesions at birth should also be recorded, as it has already been shown that the number of lesions is linked to the incidence of neurologic complications (3). Current classification in our practice is 0–10 (exact number should be noted), 10–20, 20–50, 50–100, 100–200, and >200 lesions. The largest lesion should then be classified as usual with respect to Projected Adult Size. The importance of the classification being based on the clinical picture at birth is that this condition evolves variably with age, with some patients never developing further nevi and others developing large numbers for many years. New counts/estimates of the number of lesions should be made at each follow-up.

Traditionally the term ‘multiple CMNs’ has been reserved for cases where no individual lesion is larger than the others. Under this new classification these cases would remain part of the larger group of multiple CMNs and would be identified by number and size of lesions.

The proposed correction of terminology may improve reproducibility between clinicians by encouraging more accurate recording of the number and size of lesions at a standardized age, and may therefore lead to an improvement in our ability to identify those at highest risk of complications.
